# Evaluating the detection ability of a range of epistasis detection methods on simulated data for pure and impure epistatic models

**DOI:** 10.1371/journal.pone.0263390

**Published:** 2022-02-18

**Authors:** Dominic Russ, John A. Williams, Victor Roth Cardoso, Laura Bravo-Merodio, Samantha C. Pendleton, Furqan Aziz, Animesh Acharjee, Georgios V. Gkoutos

**Affiliations:** 1 Institute of Cancer and Genomic Sciences, Centre for Computational Biology, University of Birmingham, Birmingham, United Kingdom; 2 Institute of Translational Medicine, University of Birmingham, Birmingham, United Kingdom; 3 NIHR Surgical Reconstruction and Microbiology Research Centre, University Hospital Birmingham, Birmingham, United Kingdoma; 4 MRC Health Data Research UK (HDR UK) Birmingham, United Kingdom; 5 NIHR Experimental Cancer Medicine Centre, Birmingham, United Kingdom; 6 NIHR Biomedical Research Centre, University Hospital Birmingham, Birmingham, United Kingdom; Soongsil University, KOREA, REPUBLIC OF

## Abstract

**Background:**

Numerous approaches have been proposed for the detection of epistatic interactions within GWAS datasets in order to better understand the drivers of disease and genetics.

**Methods:**

A selection of state-of-the-art approaches were assessed. These included the statistical tests, fast-epistasis, BOOST, logistic regression and wtest; swarm intelligence methods, namely AntEpiSeeker, epiACO and CINOEDV; and data mining approaches, including MDR, GSS, SNPRuler and MPI3SNP. Data were simulated to provide randomly generated models with no individual main effects at different heritabilities (pure epistasis) as well as models based on penetrance tables with some main effects (impure epistasis). Detection of both two and three locus interactions were assessed across a total of 1,560 simulated datasets. The different methods were also applied to a section of the UK biobank cohort for Atrial Fibrillation.

**Results:**

For pure, two locus interactions, PLINK’s implementation of BOOST recovered the highest number of correct interactions, with 53.9% and significantly better performing than the other methods (*p* = 4.52*e* − 36). For impure two locus interactions, MDR exhibited the best performance, recovering 62.2% of the most significant impure epistatic interactions (*p* = 6.31*e* − 90 for all but one test). The assessment of three locus interaction prediction revealed that wtest recovered the highest number (17.2%) of pure epistatic interactions(*p* = 8.49*e* − 14). wtest also recovered the highest number of three locus impure epistatic interactions (*p* = 6.76*e* − 48) while AntEpiSeeker ranked as the most significant the highest number of such interactions (40.5%). Finally, when applied to a real dataset for Atrial Fibrillation, most notably finding an interaction between *SYNE2* and *DTNB*.

## Introduction

To gain a better understanding of the underlying mechanisms that govern disease pathophysiology and pathobiology, genetic studies have been carried out at increasing volume and across large populations [1, 2]. With the promise of personalized medicine taking advantage of polygenic risk scores (PRS) for the early detection of an individual’s predisposition for certain disorders and patient specific genome-based personalized treatment development, this data holds great promise [3–5]. However, the initial hopes of Genome-Wide Association Studies (GWAS) contributing to major breakthroughs in our understanding of disease mechanisms failed to materialize. This was due to our inability to reliably identify genetic drivers, commonly referred to as the missing heritability challenge. GWAS studies, though frequently able to discover many associated genetic loci, typically recover loci with small effect sizes attributable only to a small fraction of the phenotypic variability expected. Nevertheless, similar approaches have been successful in recapturing much higher heritability estimates in twin studies [6]. Possible explanations for this shortfall have been centred around three general domains—aetiology being driven by a wide number of genes and variants, with many not captured or deemed significant, causative substitutions, indels or structural variations not being identified by the study design and the effects of epistatic genetic interactions not being identified [7, 8].

**Fig 1:**
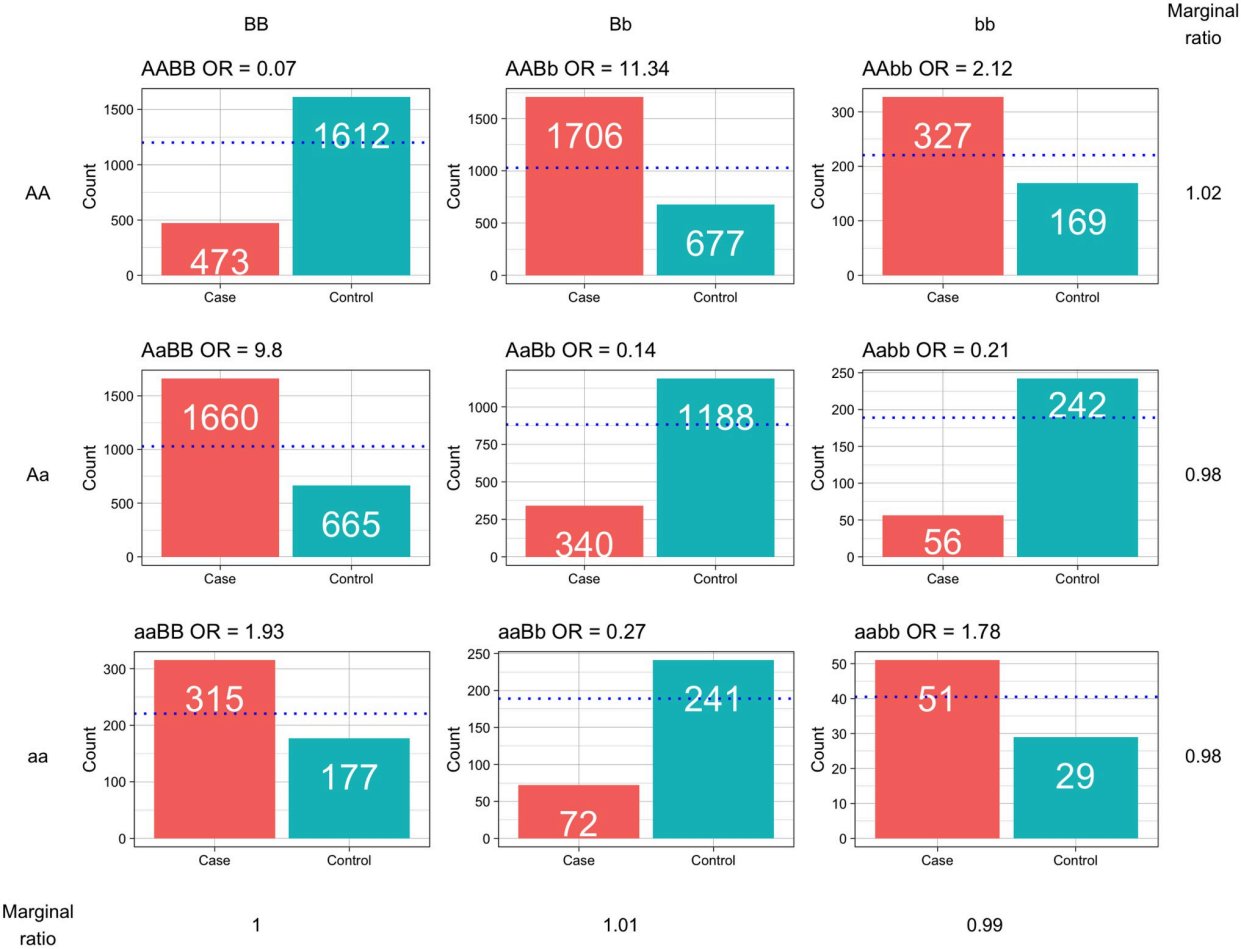
An example of the distribution of cases and controls across all possible genotypes as a result of an epistatic interaction for two loci (note that y-axes are different scales). To the right and below are the marginal effects, shown as a ratio of cases to controls. This is an example of a pure interaction because these marginal effects are very minimal. The blue dotted line indicates numbers expected under the Hardy-Weinberg Equilibrium, with capital genotypes representing the major allele and lower case the minor allele.

Epistasis is the phenomenon in which two genes interact to affect the expression of a particular phenotype, with the interaction distinguished from a simple additive effect of the joint individual genetic effects [9, 10]. Typically, epistatic interactions are depicted in penetrance tables, with two loci making up a 3x3 table, depicting all possible allelic combinations and their phenotypic contributions. These interactions can theoretically be manifested over many different combinations. Considering a binary penetrance table for high-risk or low-risk genotypes, across two loci, there are 512 (2^9^) possible configurations. Albeit, symmetrical models can be removed, still leaving 50 unique conformations [11]. The number of individuals with each genotype is also approximately bound by the Hardy-Weinberg Equilibrium (HWE), so the count is governed by a function of the minor allele frequency for each locus [12]. Additionally, loci in an interaction will not necessarily have any significant effect when examined individually, with ‘pure’ epistatic models made up of loci with no main effect on their own as in Fig 1. In such cases, both loci must be considered in order to detect the interaction [13].

A number of approaches for interaction detection have been proposed. A Web of Science query for ‘epistasis detection’ methods, designed for case/control tasks, retrieved a total of 105 methods published between 2010 and the end of 2020. Of those, 59 are currently available for download and formed the basis of the methods included in this study (S4 Table in S1 File). Many different detection strategies have been implemented, with some testing all possible interactions exhaustively and others using different filtering steps to reduce the number of loci considered and as a result the number of tests required. The methods were split between statistical approaches, such as Generalized Linear Models (GLMs) and contingency tables, data mining and machine learning (ML) techniques. Chief amongst the data mining approaches are Multifactor Dimensionality Reduction (MDR) approaches, while nature-inspired algorithms are the key representatives of ML-based approaches. Key factors, such as runtime, correcting for covariates as well as detection of higher-order interactions were also considered.

Early epistasis detection approaches utilized simple statistical techniques, such as *χ*^2^ tests [14] and GLMs for assessing interactions [15, 16]. These approaches have endured and are implemented in various tools. For example, Cassi and PLINK encompass a Z-score based test, termed fast-epistasis, while adopting slightly differing logistic regression approaches, with PLINK also implementing the Wan Log-Linear method, BOOST [17–19]. Another, more recent example is the wtest R package, that uses a novel statistic approach to compare distributional differences present in the alleles of cases and controls, using a *χ*^2^ distribution [20].

MDR is a non-parametric data mining method, which accounts for all possible genotypes, for a set of loci, summarizing the best configuration combinations for cases and controls splits. It is in essence a naive Bayes approach, with genotypes forming the parameters of a probabilistic classifier [21–23]. This modelling technique is flexible and can consider confounding factors as well as non-linear models, the latter of which renders it advantageous over GLMs. A key part of the methodology is the training and testing, with a cross validation phase. Different MDR methods have been implemented using approaches, such as incorporating odds ratios [24], fuzzy set theory [25, 26] or using a support vector machine in SVM-MDR [27].

Nature-inspired algorithms aim to mimic attributes from naturally occurring phenomena and have been applied to the challenge of efficiently identifying epistatic relationships. Ant Colony Optimization (ACO) algorithms, arguably the most prominent representatives of this type of approaches, employ simulated ants that randomly explore data to identify the most efficient path to the ‘food’, represented here by the division of cases and controls by an interaction. The ants share information and utilize algorithms to make probabilistic decisions in a network of nodes, reinforcing successful decisions by leaving ‘pheromones’ [28]. Strategies for directing ants commonly use contingency table methods, logistic regression, information theory and Bayesian networks or combinations of these in stages [29]. An early ACO algorithm example is AntEpiSeeker [30], that uses a two-stage approach with *χ*^2^ tests. More recent implementations include MACOED [31], combining the Akaike Information Criterion with a logistic regression variant and epiACO [32] that uses mutual information and Bayesian network methods. Further nature-inspired algorithm examples include the particle swarm optimization (PSO) methods [33] as well as genetic algorithms [34].

A diverse selection of other approaches have also been applied to the problem of finding epistatic interactions. Prominent examples include the approaches proposed Goudey et al, such as the gain in sensitivity and specificity (GSS) method [35, 36]. This approach employs Receiver Operating Characteristic (ROC) metrics to assess prediction performances between the interaction model and that of the two loci individually. It measures the difference in the area under a curve of all nine possible genotypes from two loci against the combined area under the curve of the two loci considered individually, giving six alleles total to be assessed.

Another interesting approach is SNPRuler, which generates association rules from combinations of loci and their genotypes using a derived statistic [37]. Also, of note are tools developed in order to harness computational resources efficiently, reducing runtime and memory requirements, such as MPI3SNP. This algorithm can be run in parallel on multiple CPUs or on GPUs for three locus interactions. The genotypes of the individuals are represented in a bitwise fashion and mutual information is calculated [38].

With such a variety of algorithms available, it is important to differentiate which will effectively discover epistatic interactions. Ibrahim et al [39] compared three algorithms, SNPRuler, SNP Harvester and Ambience using large simulated datasets and multiplicative impure epistatic interactions. They reported that none of the algorithms consistently identified the interactions but identified Ambience to be the most robust and SNPRuler to have the most power, particularly across higher order interactions taking into account more than two loci. Chatelain et al [36] compared fastepi, GBOOST, SHEsisEpi, DSS and IndOR using four impure epistatic models. They found that DSS and GBOOST were the most powerful methods, with the former being preferential in the presence of limited linkage disequilibrium (LD), for example as a result of pruning variants in tight LD. Finally, Alchamlat et al compared MDR, BOOST, BHIT, KNN-MDR, MegaSNPHunter and AntEpiSeeker using pure interactions generated from real data, reporting KNN-MDR to be the most powerful approach [40].

The purpose of this study is to provide a clear, objective, comparison between some of the most prominent epistasis detection methods in a variety of different scenarios. Since there are many different approaches available, the aim is to find the optimal method and rationalize its selection criteria. With so many possible combinations of loci, being able to confidently assert interactions found will cater for the inclusion of epistasis analysis in GWAS studies and uncover a portion of the missing heritability. To account for this, the different scenarios employed in this study have been categorized based on different underlying genetic conditions, incorporating pure and impure epistasis.

## Materials and methods

### Study design

Since the objective of this study was to differentiate the ability of various tools to correctly predict epistatic interactions between two or three loci, a number of detection methods were selected, based on their merit or the type of methodology they adopted. Several tools were considered that have been reviewed in previous studies. This included SNPRuler as considered by Ibrahim et al [39], DSS and BOOST of Chatelain et al [36] and KNN-MDR as used by Alchamlat et al [40]. However, due to lack of availability two substitutions were made, with GSS used in place of DSS and KNN-MDR with a different MDR.

The methods chosen were assessed based on their ability to uncover specific interactions. Consequently, approaches that ranked features, such as the Relief-based methods (e.g. multiSURF and TuRF) were not considered [41]. Tools were selected as representatives of common statistical approaches, such as logistic regression (e.g. Cassi and PLINK’s epistasis) as well as of novel statistics approaches, for example wtest. Data mining approaches are represented by SNPRuler, MDR and GSS that have previously demonstrated efficacy. Nature-inspired algorithms, primarily represented by ACO-based approaches, were also considered. Included were AntEpiSeeker [30], the most cited method in this domain, as well as epiACO [32], a representative of more recent approaches. Finally, CINOEDV [33] was considered as an alternative type of swarm intelligence, utilizing a PSO framework.

All tools were assessed based on simulated data, generated in a reproducible way, with a given set of parameters selected to differentiate between tools performances. For pure epistatic models, GAMETES was used to simulate models with different heritability and detection difficulty settings. Furthermore, five impure epistatic models, conforming to specific genotypes, were simulated using the EpiGEN tool. These models were situated in a LD structure for the noise loci. All scenarios have been generated for both two locus and three locus interactions and thirty replicates per scenario, with the aim of producing a robust testing strategy. Furthermore, these data can be used for future comparisons. The construction of these datasets as a testing regimen aims to cover, therefore, both marginal and non-marginal epistatic models across a range of detection difficulty degrees. The models used for testing impure epistasis, shown in Table 2, have been chosen based on penetrance tables from Evans et al [11]. In the case of joint-dominant, joint-recessive and modular forms, these have been included to provide biologically-likely scenarios. In order to broaden the detection challenge, diagonal and X-OR models were also tested to provide a more complex, but plausible, genetic landscape.

The comparison between the tools’ performance was dependent on their ability to identify the ground-truth epistatic interactions. Since they were assessed across a range of potential interactions, a ranked list of identified interactions for each tool was created. The rank of the True Positives, in each analysis, were retained when their ranked position was ≤50. When any True Positive was ranked position >50 or not detected, it was assigned a rank of 51. This ensured that any tool detecting a small number of correct interactions, ranked highly, was not highlighted as outperforming other tools that were consistently identifying a higher number of correct interactions that were ranked at different levels. Due to the resulting non-normal distribution, a lower tailed Mann-Whitney U test was used to compare each tool against the distribution of all the other tools combined, resulting into a p-value.

All experiments were carried out using the University of Birmingham’s BlueBEAR HPC service, which provides a High-Performance Computing service to the University’s research community. See http://www.birmingham.ac.uk/bear for more details. We have made the code and the settings applied available on GitHub at: https://github.com/gkoutos-group/EpistasisDetection. The versions of all software used in this study are provided in S3 Table in S1 File.

### Data generation

There are a number of challenges related to generating simulated epistasis datasets. From a biological perspective, in the genetic environment alleles loosely conform to HWE, which is a function of the Minor Allele Frequency (MAF) and are anchored locally in a LD structure. Finally, any interaction involved in causation of a phenotype will explain a quantity of the trait’s heritability, but likely leave much unexplained. Further practical challenges relate to runtime, efficiency and scalability. There are at least seven different tools available for generating simulated epistasis datasets [42]. In our study, we used GAMETES to simulate pure epistatic models and EpiGEN to create impure, defined models within LD.

GAMETES randomly generates pure epistatic models using a ‘Sudoku’ method. Similar to the puzzle game, in which rows and columns must hold a set of numbers. Here, the rows and columns of the 3x3 allelic table (in the case of two locus interactions) must have equal numbers of cases and controls. As any genotype is generated, there are constraints placed on the numbers that can appear at each other genotype. This is additionally determined according to the HWE distribution and heritability explained by the interaction [43]. EpiGEN works differently, with functionality to generate specific epistatic models, either using in built settings or via customizable model files. It makes no attempt to mask the main effects of the individual loci and so creates impure epistatic models. These are placed within a genetic context, including a simulated LD structure, based on HapMap3 data. All epistatic models from both tools were accompanied by a fixed number of randomly generated noise loci. These had a range of MAFs and conformed to HWE. They are intended to provide True Negative outcomes to differentiate the ability of the tools tested. Both pieces of software are free to use and require no proprietary software to operate [42].

We used GAMETES to configure the MAF and the heritability of the modelled interactions. The MAF selected was 0.4 in order to better populate each genotype, especially interactions with more minor alleles. Heritability is a measure of the variability attributable to the phenotype, in this case, the interaction. In our study, it was set at 0.02, 0.01 and 0.005 so to adjust the interaction effect sizes. Additionally, GAMETES provides a metric termed the Ease of Detection Measure (EDM), which indicates an assessed level of difficulty for a tool to detect the interaction. GAMETES randomly generates a set number of models, ranks them according to their EDM and returns a user-defined number of models by percentile. Here, three models were requested, the easiest disregarded and the remaining two used for testing [13, 44]. Noise loci were randomly generated with MAFs between 0.05 and 0.5, with each dataset consisting of 1,000 cases and 1,000 controls. Thirty replicates were made for each scenario with randomly generated differences. This procedure was carried out for second and third-order interactions. The settings applied in our experiments are summarized in Table 1.

**Table 1:** Configurations used for GAMETES generated models. In all datasets there are 1,000 cases and controls and 30 replicate.

Loci in Interaction	Heritability	Ease of Detection Measure	Total loci
2	0.02	2	2500
2	0.01	2	2500
2	0.005	2	2500
2	0.02	1	2500
2	0.01	1	2500
2	0.005	1	2500
3	0.02	2	500
3	0.01	2	500
3	0.005	2	500
3	0.02	1	500
3	0.01	1	500
3	0.005	1	500

EpiGEN was used to generate a corpus of synthetic genetic data based on 2000 samples modelled on Chromosome 22. Several models were included, based on possible epistatic combinations as detailed in Evans et al [11]. Models were built with a number of modes of epistatic interaction, namely joint-recessive, joint-dominant, modular, diagonal, and XOR (Table 2). This was achieved by altering the case-control ratio at the genotypes involved in the interaction, changing the penetrance. Exploratory data analysis was used to find a range of ratios that demonstrated a differentiation between the tools being tested. Noise loci made the feature space up to 500 in total and were limited to MAFs between 0.05 and 0.5. The datasets were set to aim for 1,000 cases and 1,000 controls, however these numbers did fluctuate to fit the models. Thirty replicates were made for each scenario with randomly generated differences. This procedure was repeated for third-order interactions, except with a feature space of 100 loci, including three interacting loci and 97 noise loci. For penetrance tables, see S5 Table in S1 File.

**Table 2:** EpiGEN Penetrance models with capital genotypes as the major allele.

**Joint Dominant**	**AA**	**Aa**	**aa**	**Joint Recessive**	**AA**	**Aa**	**aa**
**BB**	0	0	0	**BB**	0	0	0
**Bb**	0	1	1	**Bb**	0	0	0
**bb**	0	1	1	**bb**	0	0	1
**Modular**	**AA**	**Aa**	**aa**	**XOR**	**AA**	**Aa**	**aa**
**BB**	0	0	0	**BB**	0	0	1
**Bb**	0	0	1	**Bb**	0	0	1
**bb**	1	1	1	**bb**	1	1	0
**Diagonal**	**AA**	**Aa**	**aa**				
**BB**	1	0	0				
**Bb**	0	1	0				
**bb**	0	0	1				

The data were converted to various required formats using PLINK and R. The versions of all tools used in this study are detailed in S3 Table in S1 File. Default settings were used unless stated otherwise or expanded upon.

### Atrial fibrillation

The detection tools were employed to identify potential interactions associated to Atrial Fibrillation (AF) using patients selected from the UK biobank cohort [45]. This presents a real-world application of these methods, with the assessment of the validity of the results conducted using *a priori* knowledge. However, it also functions as a limited experiment into possible interactions present in AF.

There are a number of considerations when applying these tools to real data. The methods tested, as a group, have a number of limitations. These include memory use, runtime, inability to deal with missing values and a lack of facilities to account for environmental factors, as such requiring careful selection of samples. As a result, the cohort and number of loci considered was filtered to work within the scope of these tools. UK biobank contains genetic and phenotype data for 486,445 individuals, including 33,492 reported to have AF and 93,095,623 genotyped and imputed loci. Individuals were removed if they had been flagged as outliers for genetic missingness and heterozygosity, had a sex chromosome mismatch or evidence of sexual aneuploidy. Only Caucasians were included in the study and patients were excluded if they had a second degree relative or closer participating in the UK biobank cohort, leaving 335,400 samples. This included 23,178 individuals remaining with AF who were paired with an equal number of controls that had no diagnosis for AF, using random sampling. This left a final cohort size of 46,356. Loci were included if they had an imputation INFO score of 1 and a MAF of at least 0.495, leaving 2,478 loci. After removing those with missing values, 1592 loci remained. By using a high MAF, it ensures that under HWE each genotype is represented by many samples, thereby increasing the statistical power of the assessment. Gene annotations were derived from Variant Effect Predictor [46]. To assess if interacting genes are over-represented for Gene Ontology classes, they profiled using Bonferroni correction in gProfiler [47]. STRING was used for protein-protein interactions, keeping default settings with interactions >0.400 being significant [48].

For details of each algorithm tested, please see Supplementary Methods.

## Results

**Fig 2:**
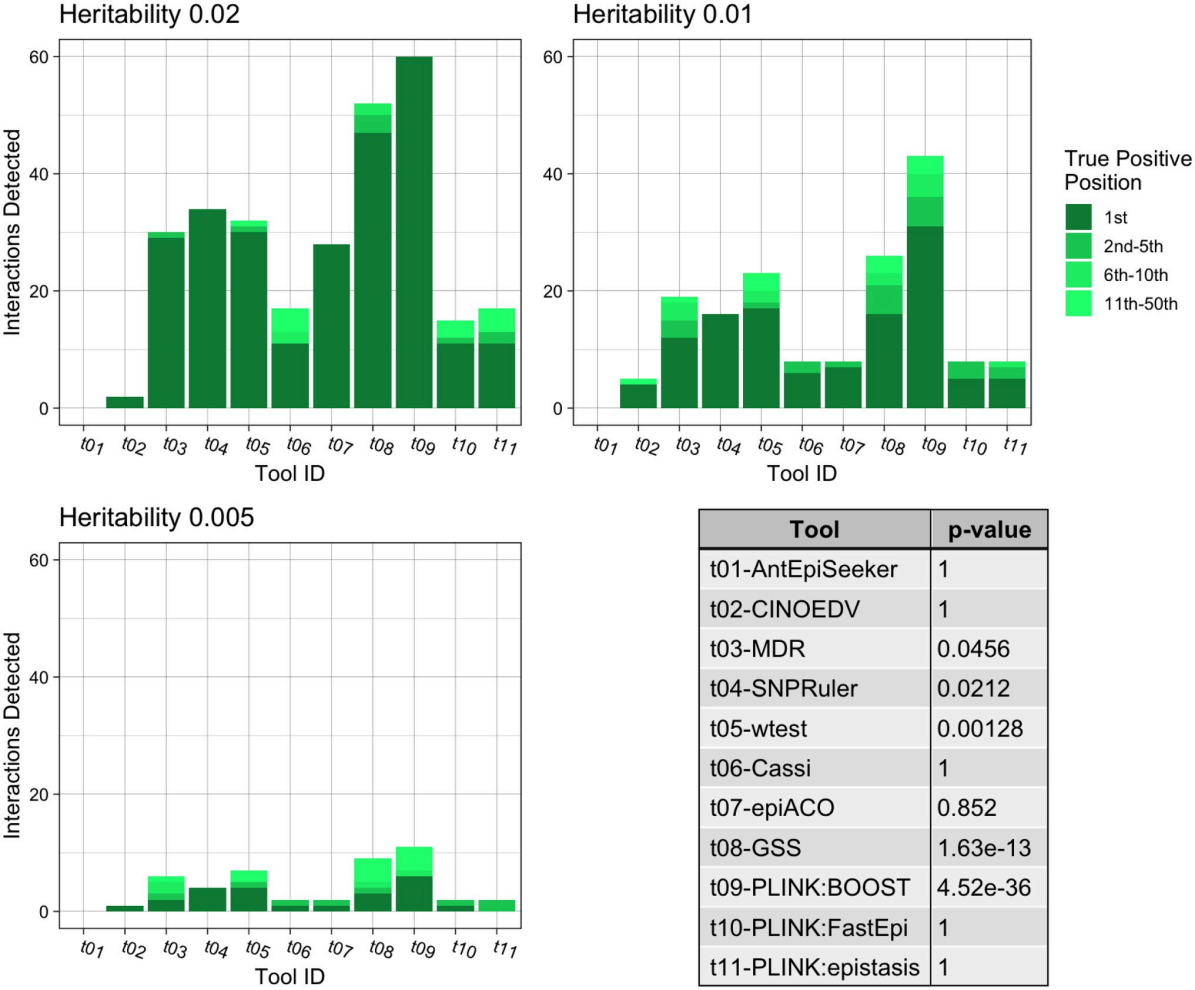
Summary of the pure epistasis results for second order interactions. The three bar charts show the number of True Positive interactions discovered and the position that the algorithm ranked it amongst combinations with noise loci. Each chart shows a different heritability for the interaction, with higher heritability explained making it more prominent against random noise. The table shows the results of a Mann-Whitney U Test comparing the non-normal distribution of True Positive ranks for a single tool against the distribution of true positive ranks for all other tools.

**Fig 3:**
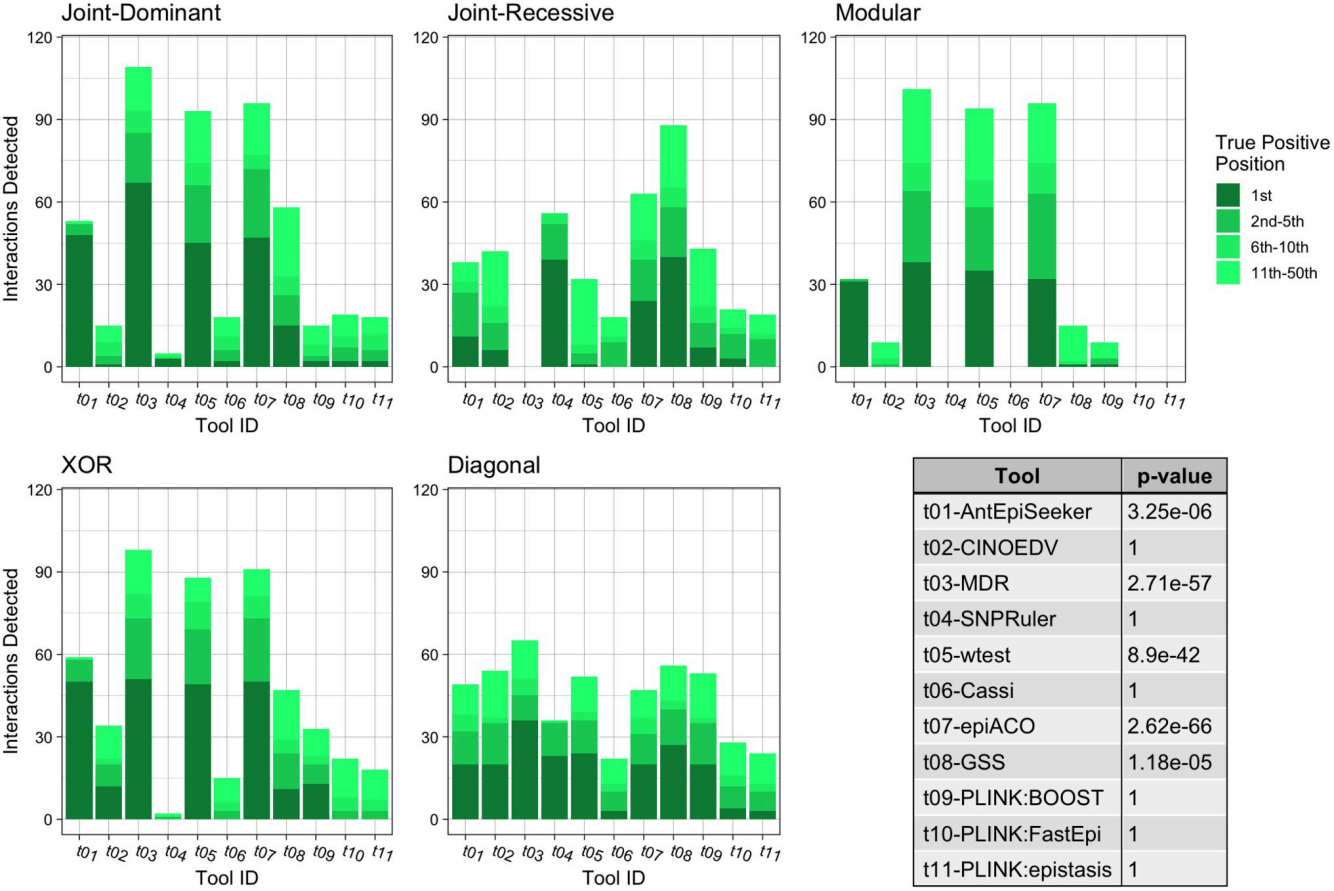
Summary of the impure model results for second order interactions. Each bar chart shows the number of True Positive interactions discovered and the position that the algorithm ranked it amongst combinations with noise loci. Each chart shows a different interaction models (see Table 2). The table shows the results of a Mann-Whitney U Test comparing the non-normal distribution of True Positive ranks for a single tool against the distribution of True Positive ranks for all other tools.

**Fig 4:**
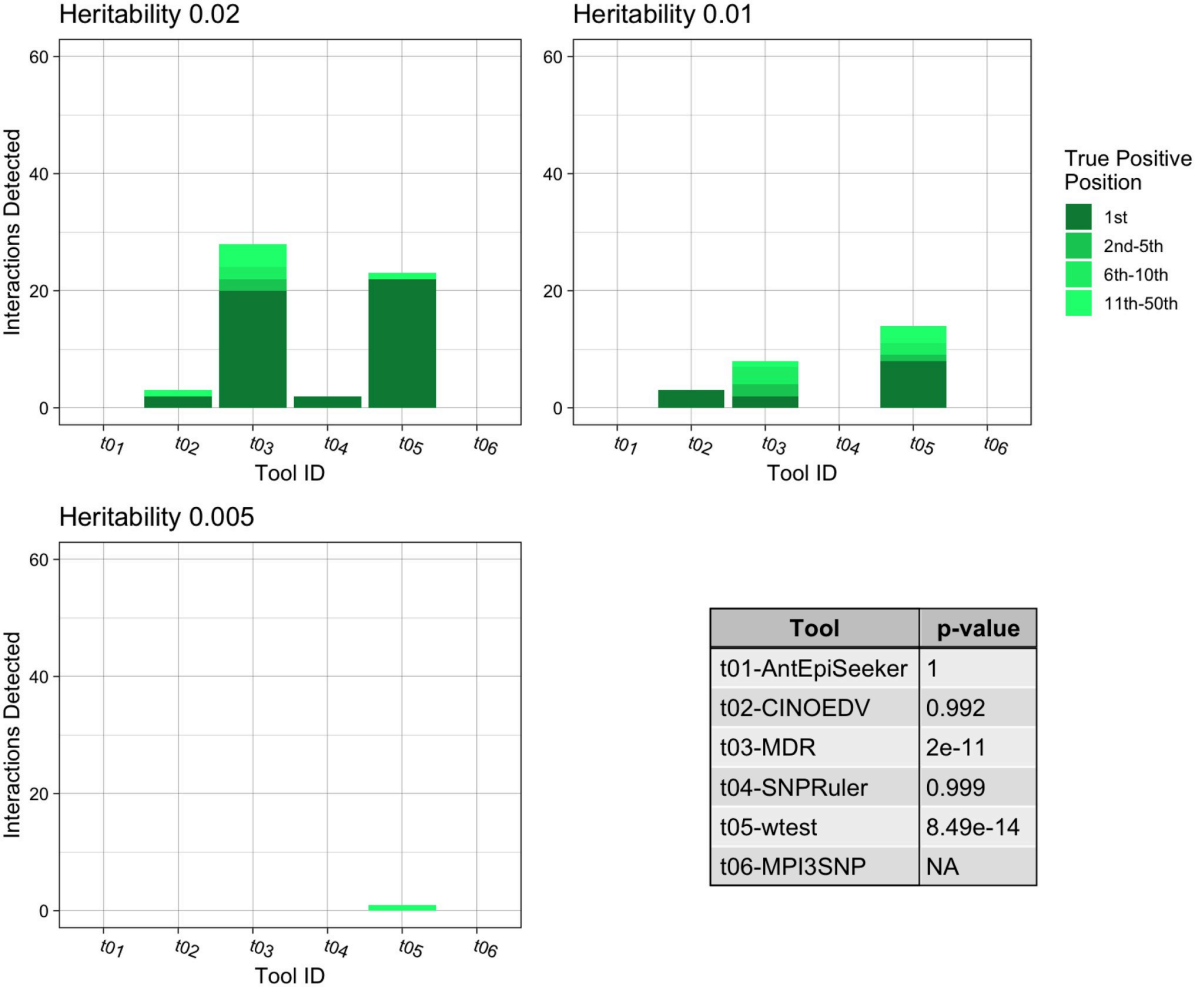
Summary of the pure epistasis results for third order interactions. The three bar charts show the number of True Positive interactions discovered and the position that the algorithm ranked it amongst combinations with noise loci. Each chart shows a different heritability for the interaction, with higher heritability explained making it more prominent against random noise. The table shows the results of a Mann-Whitney U Test comparing the non-normal distribution of True Positive ranks for a single tool against the distribution of True Positive ranks for all other tools.

**Fig 5:**
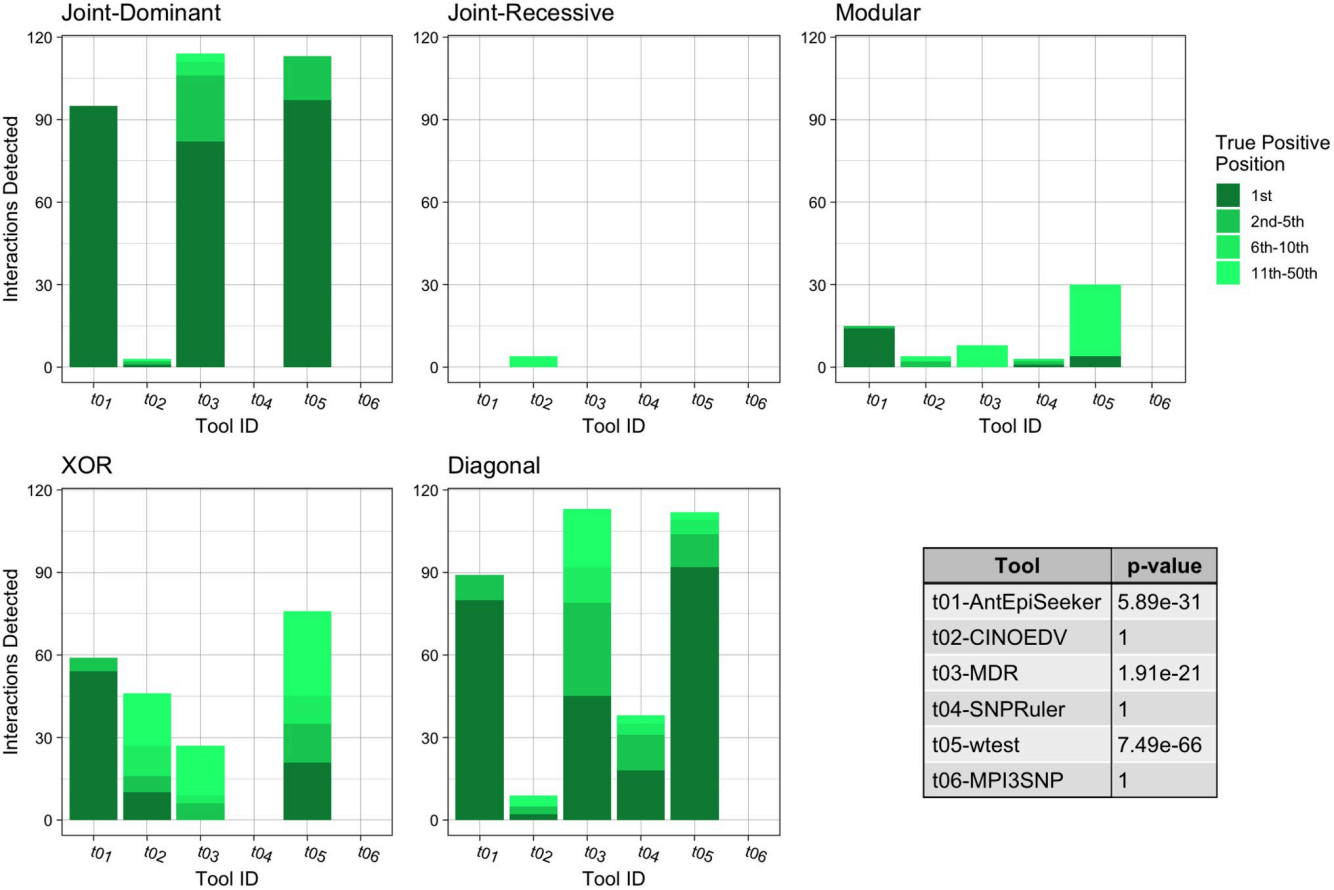
Summary of the impure model results for third order interactions. Each bar chart shows the number of True Positive interactions discovered and the position that the algorithm ranked it amongst combinations with noise loci. Each chart shows a different interaction models (see Table 2). The table shows the results of a Mann-Whitney U Test comparing the non-normal distribution of True Positive ranks for a single tool against the distribution of True Positive ranks for all other tools.

Figs 2–5 present each tool’s rank of the simulated epistatic interaction. This is limited to a rank of 50, with those interactions, which were not detected or ranked lower than 50, represented as translucent points at a provisional rank of 51. Since some methods exhaustively test all combinations, it is fair to introduce a limit beyond which a discovery is considered as ‘not detected’. Each plot represents a different set of parameters depending on the way the data were generated. For pure interactions, the graphs are split by heritability, a measure of how penetrant the interaction, whilst for the impure epistatic datasets they are split into different scenarios based on defined models. There are also different levels of detection difficulty for each model. Pure interactions are assessed by the simulation software, while impure interactions’ detection is based on the fraction of cases and controls that have the affected genotypes. For each experiment, thirty tests were carried out per set of parameters, with random differences between them. Experiments were carried out for both second-order and third order interactions, including interactions of two loci and three loci respectively, as shown below.

### Second order interactions

In the case of pure epistatic interactions (Fig 2), the PLINK implementation of BOOST exhibited the most effective performance (*p* = 4.52*e* − 36), identifying all the interactions at a heritability of 0.02, with 71.7% and 18.3% of the interactions correctly identified for 0.01 and 0.005 heritability levels respectively. GSS achieved the second-best performance (*p* = 1.63*e* − 13) followed by wtest, SNPRuler and MDR. Looking specifically at the correct interaction being top ranked, BOOST found 53.9% of the true interactions, followed by GSS, SNPRuler and wtest.

When assessing impure two locus models of epistasis (Fig 3), for all models except from the joint-recessive model, MDR exhibited the best performance (*p* = 6.31*e* − 90), identifying between 54.2% and 90.9% of interacting loci. However, for the joint-recessive model, this method has the lowest performance, failing to detect any of the interactions. In this case, GSS correctly identified most of the interacting loci, demonstrating significantly superior detection ability (*p* = 1.04*e* − 26). Notably, across all models, epiACO achieved the highest mean proportion of identified interactions at 65.5% (*p* = 2.62*e* − 66) compared to MDR at 62.2% (*p* = 2.71*e* − 57), followed by wtest with a 59.8% discovery (*p* = 8.90*e* − 42). Finally, for ranking the correct interaction first, MDR exhibited the best performance, identifying 32.0% of the correct interactions as most likely, followed by epiACO, AntEpiSeeker and wtest.

### Third order interactions

Some of the tools assessed cannot identify higher order interactions and as such were not included in the following experiments. Fig 4 presents the tools’ interaction detection performances for pure epistatic three-locus models. wtest identified 21.1% of the correct interactions (*p* = 8.49*e* − 14), marginally better than MDR (*p* = 2*e* − 11), which retrieved 20.0%. Furthermore, wtest identified 17.2% of the correct interactions as most important, compared to 12.2% for MDR.

For the case of impure epistatic models (Fig 5), wtest detected the highest number of correct interactions (*p* = 6.76*e* − 48), retrieving 56.7%, followed by MDR with 44.3% (*p* = 2.3*e* − 31) and AntEpiSeeker with 43.0% (*p* = 2.21*e* − 18). In terms of identifying the correct interaction at the highest rank, AntEpiSeeker found 40.5% of such interactions, followed by wtest with 35.7% and MDR with 21.2%. Similar to the previous experiments, the joint recessive dataset was the least well identified, with CINOEDV achieving the best performance with four accurate hits, while the others methods failed to detect any interactions.

Considering the run-time of each tool (Table 3), PLINK was the fastest for the two locus problems, its implementations of BOOST and Fast Epistasis necessitated roughly the same time with different numbers of features. All other tools required more time with additional features, apart from CINOEDV which unexpectedly required less time for greater numbers of features. However, this appears to be due to a small number of outliers that took up to 45 minutes to complete. GSS exhibited the slowest performance, taking over 46 hours to assess 2500 features. When assessing three locus problems, MPI3SNP and SNPRuler exhibited the fastest performance, with SNPRuler being fractionally slower with fewer features but requiring almost 50.0% less time for the larger feature set. The tools with the slowest performance were CINOEDV and wtest, with the latter expending over 14 hours to assess a single dataset. Notably, AntEpiSeeker appears to have performed the three locus problem quicker than two locus problems, but in fact different parameters were used for both datasets as a result of the differing numbers of features.

**Table 3:** Average time taken in minutes/RAM used in MB per method at different numbers of loci for a di-locus or tri-locus search. *AntEpiSeeker performed with slightly different settings in three-locus experiments (see Methods).

	Two Locus Detection		Three Locus Detection	
Tool	500 loci	2500 loci	100 loci	500 loci
AntEpiSeeker	1.89/6	6.35/16	1.32*/6	1.79*/9
CINOEDV	16.20/205	15.70/272	48.18/211	422.18/1346
MDR	0.26/1	6.01/291	0.37/1	22.59/2092
SNPRuler	0.34/1	0.66/306	0.56/1	0.46/2
wtest	0.41/1	7.47/2096	6.73/199	851.33/14302
MPI3SNP	-/-	-/-	0.22/1	1.23/69
Cassi	0.50/1	1.76/156	-/-	-/-
epiACO	13.20/665	20.85/697	-/-	-/-
GSS	29.45/682	2791.15/843	-/-	-/-
PLINK				
Fast Epistasis	0.23/1	0.21/1	-/-	-/-
PLINK BOOST	0.22/1	0.22/1	-/-	-/-
PLINK Epistasis	0.25/1	2.40/1	-/-	-/-

The memory requirements (Table 3) demonstrate a marked increase with the number of features and how many loci are involved in the interaction. wtest is the most memory intensive tool, necessitating over 14 GB of RAM for the 500 variable, three locus interaction tasks. The PLINK implementations and AntEpiSeeker demonstrated particularly efficiency.

### Demonstrative second order interactions in atrial fibrillation

Two-locus interaction analysis was performed on UK biobank participants with and without AF (Table 4). AntEpiSeeker, CINOEDV and epiACO all found a partner for rs730072, with CINOEDV finding a relationship with rs4668136, as opposed to AntEpiSeeker and epiACO, which both identified rs1152591. MDR and wtest also found a potential interaction with rs1152591, but instead the other locus was rs3792234. GSS and BOOST also found the same pair of SNPs most explanatory, with a relationship predicted between rs9346918 and rs4342945. Similarly, Cassi and epistasis found the same pairs in rs1608994 and rs3809775. This SNP set was significantly over-enriched for the Gene Ontology terms, zinc ion binding (*p* = 4.04*e* − 2) and RNA polymerase II transcription regulator complex (*p* = 4.03*e* − 2). Moreover, there is evidence in the STRING database for protein interactions [48] showing that homologs of *DTNB* and *SYNE2* in *Mus musculus* are co-expressed and interact in various assays, an interaction that was found by two independent tools. This could potentially be a fruitful line of enquiry given that *SYNE2* is known to be associated with AF [49]. Additionally, *LRP2*, which epistatically interacts with *DTNB*, has been linked to arrhythmias in multiple conditions from genomic screens and proteomic assays [50, 51].

**Table 4:** Epistatic interactions predicted by each tool as most important for Atrial Fibrillation. An * denotes that the SNP was found to be intergenic and this is the nearest gene.

Tool	SNP1	Gene1	SNP2	Gene2
**AntEpiSeeker**	rs730072	*DTNB*/*ARNILA*	rs1152591	*SYNE2*/*ESR2*
**Cassi**	rs1608994	*MSR1*	rs3809775	*HOXB8*/*HOXB9*
**CINOEDV**	rs730072	*DTNB*/*ARNILA*	rs4668136	*LRP2*
**epiACO**	rs730072	*DTNB*/*ARNILA*	rs1152591	*SYNE2*/*ESR2*
**GSS**	rs9346918	*PRKN*	rs4342945	*PPP2R2D*
**MDR**	rs3792234	*STON1*	rs1152591	*SYNE2*/*ESR2*
**PLINK:epistasis**	rs1608994	*MSR1*	rs3809775	*HOXB8*/*HOXB9*
**PLINK:FastEpi**	9:140746691	*EHMT1*	rs56018060	*CA12*/*LINC02568*
**PLINK:BOOST**	rs9346918	*PRKN*	rs4342945	*PPP2R2D*
**SNPRuler**	rs6754266	LOC105373398*	rs12627212	*RUNX1*
**wtest**	rs3792234	*STON1*	rs1152591	*SYNE2*/*ESR2*

## Discussion

This study has systematically assessed the performance of several state-of-the-art tools, including popular implementations, such as logistic regression, swarm intelligence and multifactor dimensionality reduction. The evaluation of their performance was carried out for two key categories of epistasis, namely pure epistatic interactions with no main effect, which were randomly distributed and impure epistatic interactions with some main effect, that conformed to several set epistatic models. This evaluation strategy was repeated for both second-order and third-order interactions. The purpose of this has been to inform future research to uncover genetic interactions, reduce the impact of missing heritability and ultimately to better inform use of genetic data as we strive towards personalized medicine.

Our experiments revealed that the performance of each tool varies depending on the task that is being assessed. The pure epistatic models represent the most difficult interactions to detect, since the lack of marginal effects necessitate all loci in an interaction to be considered to identify any effect. Each locus individually will not have a main effect and as such its effect would have insignificant p-values in a standard univariate GWAS, with p-values around 1 (S1 Fig). However, the tools which detected the most pure epistatic models were not the same as those that found the most impure models, indicating that tool selection based on significance is a valid strategy.

In the case of pure two locus models, the PLINK implementation of BOOST had the best performance. It was also the fastest to complete the task (Table 3). This is perhaps expected, since PLINK is one of the first tools in this space with continuous maintenance and improvements including speed improvements, utility of bitwise operators, multithreading and other techniques. A disadvantage of BOOST lies with the lack of a function to include covariates, necessitating their correction in post-processing steps.

Since PLINK only assesses pair-wise interactions, it is not a viable option for third order interactions. For these cases, wtest detected marginally a higher number of pure epistatic interactions than MDR and ranked them first in 81.6% of instances, compared to MDR’s 61.1%. However, the runtime and memory requirements for wtest are prohibitive, especially for larger datasets. Perhaps a two stage strategy, such as using MDR to select a number of candidates, which could then be tested further with wtest, could be an efficient searching approach.

Considering impure epistatic interactions, the MDR was most successful at detecting two locus problems in all test cases, except for the joint recessive model. This type of model is the hardest to detect since the interaction only occurs at only one genotype. Since MDR considers all possible genotypes, it is likely that random fluctuations in other genotypes detract from the effect of the affected genotype, which contains the fewest total individuals under HWE. For a minor allele frequency (MAF) of 0.4, with a total of 2,000 samples, there are approximately 50 samples with this genotype in a two locus problem and 7 for three loci. This figure is then split into cases and controls, rendering distinguishing a three locus joint recessive scenario very difficult, even for the instances that all or almost all of the samples with that genotype were cases. GSS retrieved the most of the two loci cases, whilst CINOEDV found four higher order interactions. However, it is questionable how feasible searching for these interactions is without much larger sample sizes. Higher order impure epistatic models returned mixed results, with wtest again performing well with higher dimensionality but ranking fewer at first place than AntEpiSeeker. MDR was also notable for detection ability, and perhaps again a joint searching strategy could be employed to give a greater combination of speed and accuracy.

Evidently our assessment, given that it encompasses a survey of a wider range of tools than previous reviews of this nature, clearly demonstrates that there is no one best tool for all interaction types. GSS has been previously been reported as the most powerful algorithm by Chatelain et al [36] and although our results showed a similar trend, GSS was not the optimum solution for any of the scenarios we assessed, exhibiting substantially long run times and memory requirement. Alchamlat et al [40], reported MDR as the best performing tool and our results indicate that, although it did not exhibit the best performance across the datasets that we used, its performance was the most consistent. Furthermore, we found little to suggest an ensemble of tools would provide better identification of interactions. If the interaction was to be retrieved by any tool, the most proficient tool, in each scenario, was almost always the one that retrieved it and as such, multi-tool ensemble-based approaches were not found to be useful (for key features of each tool see S6 Table in S1 File).

The application of the tools to the UK biobank AF data highlighted limitations found in some of the tools, such as the inability to handle missing genotypes and the memory requirements needed to run wtest when applied to a larger dataset. Given *SYNE2* has previously been associated with AF [49], that does lend some credence to the interactions involving that gene, that was found by four tools. Interestingly, there was evidence in STRING [48] for an interaction between homologs of *DTNB* and *SYNE2*, being both co-expressed in *Mus musculus* and interacting within various assays. This perhaps supports the use of AntEpiSeeker and epiACO, however more research would need to be carried out. The experiment did demonstrate some patterns amongst the tools. Unsurprisingly, both PLINK’s epistasis and Cassi identified the same interactions, since they are both variants of logistic regression. Interestingly, there was also exact agreement between GSS and BOOST, as well as wtest and MDR and a high-level of similarity amongst the swarm intelligence methods. This indicates that perhaps some concordance between how these methods approach the data that has led them to rank highly the same interactions.

A key limitation to this paper is the focus on detection capabilities of tools at a smaller scale than a regular GWAS. The next step will involve transitioning to larger feature spaces of around 750,000 initial loci and assessing different strategies for searching efficiently, especially since the time and memory requirements for spaces of this size will be very large. This will therefore require testing strategies that can be applied at a larger scale, and is therefore beyond the scope of this paper. Using a faster more efficient tool initially, followed by a quicker more accurate one could be a potential solution. Alternatively, individual features could be ranked first using a Relief-based algorithm, such as multiSURF [41]. Furthermore, since different tools perform better for pure and impure epistatic problems, a division of the data can be made using the p-value of individual loci generated by logistic regression. Finally, this research is limited to second and third-order interactions. Further work is planned to be carried out for higher-order interactions, but establishing feasibility at lower levels has been carried out preferentially due to the power constraints which are increased at each dimension. Since the loci exist in HWE, their genotypes containing fewer samples is unavoidable, an effect that is compounded with additional dimensionality.

We note that there are a large number of epistasis detection tools in existence, and therefore it is plausible that some of them would exhibit better performances than the selection assessed here. Our aim was to provide an independent, objective performance assessment over a representative selection of available tools. Our inclusion criteria considered whether it was feasible for a tool to be assessed under our study constraints, and hence tools designed for quantitative phenotypes as well as tools that relied upon the wider genome context were not included. Examples of the latter would be Eigen-epistasis [52] that calculates eigenvectors for sections of the genome or GenEpi that uses gene boundaries to group loci [53].

The practical application of this research is dependent upon further development of these approaches in order to create workflows to find interactions at scale for many diseases and phenotypes. In the clinic, the additional genetic markers have the potential to be used in diagnostic applications and as targets for therapeutic remedies. Owing to the substantial effort expended to carry out GWAS, there is significant drive to ensure there are beneficial medical advances as a result [54, 55]. Quantifying the heritability and effect size of the interactions allows for their incorporation with known single loci involved in the disease. Inclusion of interactions within models for PRS has been shown to affect overall accuracy [56]. Additionally, *apriori* data about interactors can guide further research into particular diseases and enhance biological interpretability. Knowledge can be revealed using powerful resources, such as Reactome and KEGG datasets for pathway analysis, Gene Ontology for protein function, processes and cellular location and STRING Database for protein-protein interactions [48, 57–59].

Our approach revealed that different tools are optimum for different challenges. For detecting pure, two locus interactions, BOOST, as implemented by PLINK, was most effective, with low runtime and memory requirements. For impure epistatic interactions, MDR retrieved the highest number of correct interactions. For the more computational costly cases, MDR offers options for covariate correction. Finally, for detecting three locus interactions, wtest exhibited the best performance, albeit with the highest computation requirements amongst all tools assessed and as such, filtering steps would potentially be required that could be applied prior to its application.

## Conclusion

Through the generation of 1,560 datasets and testing of twelve tools, we have demonstrated that there are preferential selections for each problem. When detecting pure, two locus interactions, BOOST as implemented by PLINK exhibited the most effective and fastest performance with low memory requirements. For impure epistatic interactions, MDR identified the most correct interactions. While the computational requirements were higher, a notable benefit of using MDR is that it offers some facility for covariate correction. Finally, for detecting three locus interactions wtest resulted in the best performance albeit necessitating the highest amount of computation resources amongst the tools assessed, implying that potentially a filtering step prior to its application might be beneficial.

## Supporting information

S1 File (ZIP)

S1 Fig Graphical abstract. (TIF)
