# Correction: Evaluating the detection ability of a range of epistasis detection methods on simulated data for pure and impure epistatic models

**DOI:** 10.1371/journal.pone.0288416

**Published:** 2023-07-06

**Authors:** Dominic Russ, John A. Williams, Victor Roth Cardoso, Laura Bravo-Merodio, Samantha C. Pendleton, Furqan Aziz, Animesh Acharjee, Georgios V. Gkoutos

After publication of this article [1], concerns were raised regarding the methodology and statistical analyses, in particular that the results of third-order epistasis comparison of MPI3SNP with the other methods could not be replicated.

Here, the authors have provided additional information to clarify these issues:

A coding error was found when processing the results from MPI3SNP. The output file includes column numbers that are 0 indexed, however these were processed with 1 as the number for the first column in error. As a result, it was reported that MPI3SNP had not recovered any interactions. This proved to be incorrect and MPI3SNP is one of the best performing tools for three locus interactions.

The authors have provided updated Figs 4 and 5 with the correct results and provided the following corrections:

**Fig 4:**
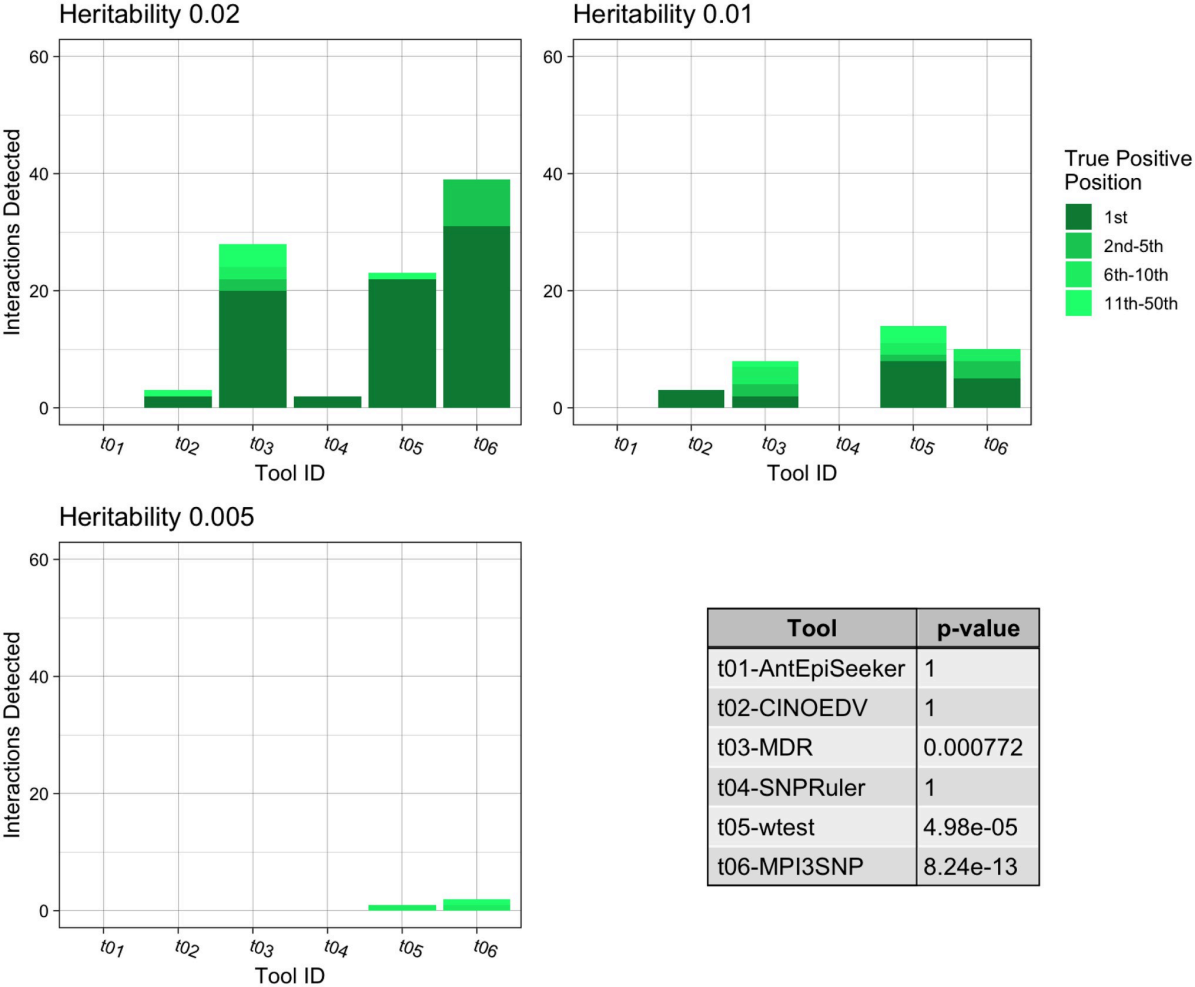
Summary of the pure epistasis results for third order interactions. The three bar charts show the number of True Positive interactions discovered and the position that the algorithm ranked it amongst combinations with noise loci. Each chart shows a different heritability for the interaction, with higher heritability explained making it more prominent against random noise. The table shows the results of a Mann-Whitney U Test comparing the non-normal distribution of True Positive ranks for a single tool against the distribution of True Positive ranks for all other tools.

**Fig 5:**
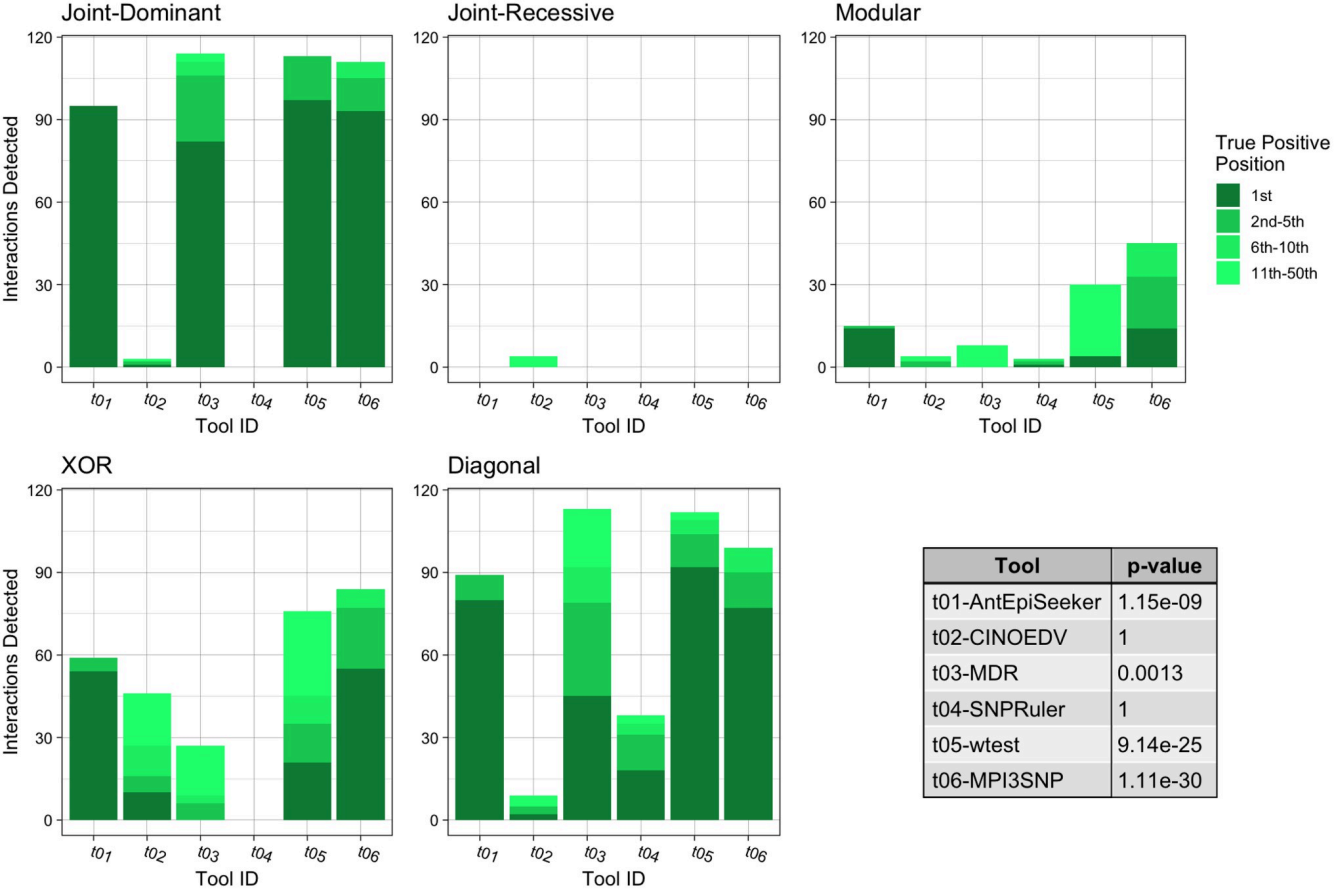
Summary of the impure model results for third order interactions. Each bar chart shows the number of True Positive interactions discovered and the position that the algorithm ranked it amongst combinations with noise loci. Each chart shows a different interaction models (see Table 2). The table shows the results of a Mann-Whitney U Test comparing the non-normal distribution of True Positive ranks for a single tool against the distribution of True Positive ranks for all other tools.

The fifth sentence of the Results subsection of the Abstract, is clarified with updated wording as follows: The assessment of three locus interaction prediction revealed that MPI3SNP recovered the highest number (28.3%) of pure epistatic interactions (*p* = 8.24*e* −13). wtest recovered the highest number of three locus impure epistatic interactions, 56.7%. However, MPI3SNP demonstrated a superior distribution of ranking (*p* = 1.11*e* −30) and required much less computational resources. AntEpiSeeker found as the most significant the highest number of simulated interactions (40.5%).

In the Third order interactions subsection of the Results, there are the following corrections:

In the second sentence of the first paragraph, the correct sentence is: Fig 4 presents the tools’ interaction detection performances for pure epistatic three-locus models. MPI3SNP identified 28.3% of correct interactions (*p* = 8.24*e* −13), followed by wtest which retrieved 21.1% (*p* = 4.98*e* −05), and MDR with 20.0% (*p* = 7.72*e* −4). Furthermore, MPI3SNP identified 20.5% of the correct interactions as most important, compared to wtest and MDR with 17.2% and 12.2%, respectively.

In the first sentence of the second paragraph, the correct sentence is: For the case of impure epistatic models (Fig 5), wtest detected one more interaction than MPI3SNP, retrieving 56.7% and 56.5%, respectively. However, the Mann-Whitney U Test demonstrates that the rank distribution of those identified by MPI3SNP was superior, achieving a *p*-value of 1.11*e* −30 compared to 914*e* −25. These were followed by MDR with 44.3% (*p* = 1.3*e* −4) and AntEpiSeeker with 43.0% (*p* = 1.15*e* −09). In terms of identifying the correct interaction at the highest rank, AntEpiSeeker found 40.5% of such interactions, followed by MPI3SNP with 39.8%, wtest with 35.7% and MDR with 21.2%.

In the Discussion section, there are the following corrections:

In the second sentence of the fourth paragraph, the correct sentence is: MPI3SNP detected a higher number of pure epistatic interactions than wtest, achieving a superior distribution of ranks when tested. Of the interactions found, those ranked first made up 72.5% for MPI3SNP, compared to wtest with 81.6% of instances.

In the eighth sentence of the fourth paragraph, the correct sentence is: Higher order impure epistatic models returned mixed results, with MPI3SNP again performing well with higher dimensionality but ranking fewer at first place than AntEpiSeeker. wtest and MDR were also notable for detection ability, and perhaps again a joint searching strategy could be employed to give a greater combination of speed and accuracy.

In the last sentence of the last paragraph, the correct sentence is: Finally, for detecting three locus interactions, MPI3SNP exhibited the best performance, with the minimal computation requirements notable for this challenge.

The last sentence of the Conclusion section is corrected to: MPI3SNP resulted in the best performance, also achieving this with minimal computational overheads.
