# Supplementary material for: Evaluating the detection ability of a range of epistasis detection methods on simulated data for pure and impure epistatic models
Source: PLoS One. 2022 Feb 18;17(2):e0263390. doi: 10.1371/journal.pone.0263390 (PMC8856572; doi:10.1371/journal.pone.0263390)
Supplement: S1 File — (ZIP) [file pone.0263390.s001.zip › SuppTab6.pdf]

Key features for each tool

Tool	Statistical Test	Exhaustive Search	Mem. Use	Higher Order	Missing Data	Covariates
AntEpiSeeker	χ^2	No	Low	Yes	No	No
CINOEDV	Co-information	Both	High	Yes	No	No
MDR	Permutation Testing	Yes	Med	Yes	Yes	Yes
SNPRuler	χ^2	No	Low	Yes	Yes	No
wtest	W-test	Yes	High	Yes	No	No
Cassi	Logistic Regression	Yes	Low	No	Yes	Yes
epiACO	Mutual Information	No	High	No	No	No
GSS	Min-Max Optimization	Yes	High	No	No	No
PLINK:BOOST	Log-Linear Regression	Yes	Low	No	Yes	No
PLINK:FastEpi	Z-Score	Yes	Low	No	Yes	No
PLINK:epistasis	Logistic Regression	Yes	Low	No	Yes	Yes
MPI3SNP	Mutual Information	Yes	Low	Yes	No	No
