# Supplementary material for: Evaluating the detection ability of a range of epistasis detection methods on simulated data for pure and impure epistatic models
Source: PLoS One. 2022 Feb 18;17(2):e0263390. doi: 10.1371/journal.pone.0263390 (PMC8856572; doi:10.1371/journal.pone.0263390)
Supplement: S1 File — (ZIP) [file pone.0263390.s001.zip › Supporting information.pdf]

Supporting information

1 Supplementary methods

Statistical approaches

One category of approaches focuses on using straight-forward statistical tests and simply test every combination exhaustively. These approaches include contingency table methods, log-linear regression and logistic regression as well as novel statistics in order to assess each potential interaction.

PLINK: fast epistasis

Three PLINK [18] epistasis detection methods were assessed. The first one, fast-epistasis, takes the number of individuals for cases and controls at all genotypes for two loci (Table S1) and condenses them to a 2 x 2 table (Table S2).

Table S1. Two locus 3 x 3 table for allelic combinations, here n is equal to the number of individuals with each genotype

Table S2. Reduced 2 x 2 table for two loci, here n is equal to the number of individuals with each genotype

Using the matrix in the Table S2, log odds and variance can be calculated as shown below, where OR is odds ratio, v is variance, and A-D refer to cells of the matrix:

$$OR = \log \frac{AD}{BC} \tag{1}$$

$$v = \frac{1}{A} + \frac{1}{B} + \frac{1}{C} + \frac{1}{D} \tag{2}$$

Lastly, the χ^2 test statistic is calculated comparing cases and controls:

$$T = \frac{(OR_{case} - OR_{control})^2}{v_{case} + v_{control}} \tag{3}$$

PLINK: BOOST

The second is an implementation of BOOST, a log-linear or Poisson regression model generating a contingency table, similar to the Table S2 but with an additional dimension to divide cases and controls, given as k. An observed count is denoted as n_{ijk} with i and j the genotype at two loci. This is the result of the random variable N_{ijk} that is assumed to follow a Poisson distribution. The probability that an observation falls into any one cell is denoted as π_{ijk}, with the sum of all π being 1. The mean is given by:

$$\mu_{ijk} = n\pi_{ijk} \tag{4}$$

The likelihood is calculated with the equation:

$$f(\mu) = \prod_{i,j,k} \frac{e^{-\mu ijk} \cdot \mu_{ijk}^{nijk}}{n_{ijk}!} \tag{5}$$

This is transformed to the log-likelihood, represented as:

$$L(\mu) = \sum_{i,j,k} [n_{ijk} \log(\mu_{ijk}) - \mu_{ijk} - \log(n_{ijk}!)] \tag{6}$$

This methodology allows for fast, exhaustive testing.

PLINK: epistasis

The final PLINK method, called simply 'epistasis' assesses each interaction using logistic regression. This uses an additive model with an extra multiplicative term to test for an epistatic interaction. The $\hat{\beta}$ for the interaction term is estimated and tested for significance. This method also allows for covariates to be adjusted for.

Cassi

Cassi [17] provides an alternative logistic regression approach to the PLINK logistic regression model. Initially, the phenotype is regressed for an additive and a multiplicative model. Then a likelihood ratio test is performed for models with and without the interaction term. Thus the deviance from the additive model is attributed to the interaction. Cassi also allows for covariates to be adjusted.

wtest

Another contingency table method is wtest [20], which can be used to test one to many loci based on k combinations of interacting loci and a binary phenotype, making a 2 x k table. The method assesses distributional differences between cases and controls. Taking the ith column of k, the numbers of n_{1i} cases and n_{0i} controls and the total number of N_1 cases and N_0 controls, the conditional probabilities can be estimated, $\hat{p}_{1i} = n_{1i}/N_1$ and $\hat{p}_{0i} = n_{0i}/N_0$. The full formula uses a χ^2 distribution with f degrees of freedom:

$$W = h \sum_{i=1}^{k} \left[\log \frac{\hat{p}_{1i}/\left(1-\hat{p}_{1i}\right)}{\hat{p}_{0i}/\left(1-\hat{p}_{0i}\right)} / SE_i \right]^2 \sim \chi^2_f \tag{7}$$

With the scalar h and f as covariance matrices of the log odds ratios, estimated from bootstrapped samples under the null hypothesis. The Standard Error is calculated as follows:

$$SE_i = \sqrt{\frac{1}{n_{0i}} + \frac{1}{n_{1i}} + \frac{1}{N_0 - n_{0i}} + \frac{1}{N_1 - n_{1i}}} \tag{8}$$

An option to filter the loci by main effect p-value is also provided. Since this option for our setting would result in all pure interactions being missed, the filter was set to include all loci with a p-value less than 1. Beyond exhaustive statistical tests, approaches have been devised to optimize a search strategy before assessing the interaction.

Swarm intelligence approaches

AntEpiSeeker

AntEpiSeeker [30] uses a standard ant colony optimization framework over i iterations, in which m ants are guided through a path of n interacting genetic loci, selected dependent on a probability density function (PDF) for each locus k. In these models, the ants leave pheromones to indicate favourable paths, which evaporate over time. The PDF is calculated as follows:

$$p_{ki} = \frac{\tau_{ki}^{\alpha} \cdot \eta_k^{\beta}}{\sum_{j=1}^{L} \tau_{ji}^{\alpha} \cdot \eta_k^{\beta}} \tag{9}$$

η^{β} represents some prior 'attractiveness' information, which is set to 1 here. As a result, the PDF represents the pheromones at locus k divided by the sum of the pheromones at each of j locus from a set of L total loci. α represents the weight of the pheromone at the locus and

β is the weight of the heuristic information. The pheromone levels are updated according to the χ^2 test score for the interacting loci, as such:

$$\tau_{k(i+1)} = \left(1-\rho\right)\tau_{ki} + \Delta\tau \tag{10}$$

Where ρ is a number between 0 and 1, representing the pheromone evaporation rate and $\Delta\tau$ is the change in pheromones at locus k at iteration i and is equal to $0.1\chi^2$. This is repeated for all m ants over i iterations.

In the documentation for AntEpiSeeker there are suggested values for most of the parameters and these were used in our experiment. However, for two parameters, iTopModel and iTopLoci, this guidance was not given. This is presumably because they are dependent on the number of loci assessed since they define the number of possible models and the number of loci with the maximum quantity of pheromones. For second order interactions they were set to 1,000 and 200 respectively. So as to reflect the smaller number of loci assessed for the third order interactions these values were set to 50 and 10.

epiACO

The epiACO [32] approach adopts the same framework as AntEpiSeeker, shown in Eq 9. However, it employs a different method for testing interactions and parameter values. The interaction test statistic used, termed the SValue, uses Mutual Information (MI), the entropies of S loci and Y phenotype, and a Bayesian metric, the K2 Score in a logarithmic form:

$$MI(S;Y) = H(S) + H(Y) - H(S,Y) \tag{11}$$

$$SValue = \frac{MI}{K2score_{log}} \tag{12}$$

There are a number of search strategies adopted in order to make the search more effective. The path selection strategy involves ants taking either a probabilistic route or a stochastic one. The probability P of ant k selecting locus i at iteration t is defined as:

$$P_k^i(t) = \begin{cases} R & q <= q_0 \\ S & q > q_0 \end{cases} \tag{13}$$

In which q is a randomly generated number from a uniform distribution of [0,1] and q_0 is the iteration divided by the total number of iterations. The probabilistic path is defined as:

$$R = \begin{cases} \frac{\tau_i(t)^\alpha \cdot \eta_i^\beta}{\sum\limits_{u \in U_k(t)} \tau_u(t)^\alpha \cdot \eta_u^\beta} & i \in U_k(t) \\ 0 & otherwise \end{cases} \tag{14}$$

where $\tau_i(t)$ is the pheromones at locus i and η_i is the heuristic information at the locus. $U_k(t)$ is the set of not-selected loci by ant k. α represents the weight of the pheromone at the locus and β is the weight of the heuristic information. The stochastic path strategy follows:

$$S = \begin{cases} 1 & i = rand(V_k(t)) \\ 0 & otherwise \end{cases} \tag{15}$$

where all loci at iteration t are sorted in descending order by pheromones, with the latter half being represented by $V_k(t)$. This allows for a wider search space at lower iteration numbers. The pheromone updating strategy at iteration i and locus k can then be defined as:

$$\tau_{i(t+1)} = (1-\rho)\tau_{i(t)} + \Delta\tau_{i(t)} + \Delta\tau^*_{i(t)} \tag{16}$$

With $\Delta\tau_{i(t)}$ a pheromone increment for an ant visiting and $\Delta\tau^*_{i(t)}$ being a bonus increment for those that belong to candidate solutions based on the S-value calculated. $\Delta\tau_{i(t)}$ is found for ant a of m total ants:

$$\Delta\tau_{it} = \sum_{a=1}^{m} \Delta\tau^a_{it} \tag{17}$$

The default settings of the epiACO implementation return only the top three interactions and, in order to make its output comparable with the other assessed methods, the code was modified to return the top 50 interactions.

CINOEDV

CINOEDV [33] uses particle swarm optimization (PSO). A number of particles are simulated and distributed across a k loci space with the aim to position themselves at the strongest interaction. After initial random placement, they adjust their velocity and position. This is dependent on shared information with nearby particles and an assessment of their local space using a novel co-information method based on entropy to assess interactions. At each iteration g, the position (S) and velocity (v) of each particle is updated:

$$\tilde{v}^{g+1}_{qk} = W^g_{qk} \cdot v^g_{qk} + c_1 \cdot r_1 \cdot \left(PS^g_{qk} - S^g_{qk}\right) + c_2 \cdot r_2 \cdot \left(GS^g_{qk} - S^g_{qk}\right) \tag{18}$$

for the qth particle. W^g_{qk} is an inertia term, which takes into account the local scores against those elsewhere. The c terms are acceleration constants, and r terms are random values between 0 and 1. *PS* and *GS* represent the particle's most favourable position it has visited and that of the whole swarm, respectively. The position is then updated as such:

$$\tilde{S}^{g+1}_{qk} = S^g_{qk} + v^{g+1}_{qk} \tag{19}$$

The result is that the particles move into groups, centred around the interactions that produce the best outcomes.

Data mining approaches

MDR

The MDR [49] algorithm splits the data into a training set and a test set at a ratio of 9:1. During the training stage, the probabilities for each genotype, at two or more loci, given the status of case or control, are calculated. The interaction is defined by the probability that each genotype is a case. The number of dimensions considered for a two locus problem is thereby reduced from nine genotypes to the product of those genotypes. This evaluation is a naive Bayes classifier, defined by distributions of cases and controls across the possible genotypes:

$$v_{NB} = \underset{v_i \in V}{\operatorname{argmax}}\, p\left(v_j\right) \prod_{i=1}^{n} p\left(a_i | v_j\right) \tag{20}$$

Where v_j is one of a set of V phenotypic classes and a_i is an attribute describing each multi-locus genotype present. The output is a binary variable ascribing the presence of a genotype associated with cases. An assessment of accuracy is carried out using the reserved test set with the mean of sensitivity and specificity:

$$Accuracy = 0.5 \times \left(\frac{TP}{TP+FN} + \frac{TN}{TN+FP}\right) \tag{21}$$

By splitting the genotypes into high-risk and low-risk genotypes, MDR reduces the multi-locus space into a binary variable and assesses the accuracy that interaction assigns true-positives and true-negatives.

GSS

Gain in sensitivity and specificity (GSS) [35] is a method which employs measures of sensitivity and specificity to compare an additive and interaction model. The additive model is calculated by assessing each of the genotypes of the individual loci for sensitivity and specificity and taking the largest area under the Receiver Operator Characteristic (ROC) curve. This is compared to the area under the ROC curve for each of the nine genotypes represented in the interaction model. Hence, any gain in these measures can be attributed to the interaction and quantified using a p-value calculated from a min-max optimization. By considering the difference between the predictive power of the loci individually against the nine possible genotypes from two loci, the sensitivity and specificity are optimized.

SNPRuler

SNPRuler [37] uses predictive rule learning to find possible epistatic interactions. Rules are generated in trees for different allelic configurations, based on classification of cases or controls. Any individual SNP can be defined multiple times for different genotypes, to account for more complex epistatic models. However, any additional rule must increase predictive power. This is assessed using a derived χ^2 measure to assess potential additional rules, which must achieve this increase to be appended to the current rule. The upper bound, UB, of the potential addition to the rule is calculated:

$$UB = \frac{(Rm - (b - \min(b, d')))^2}{(m + (b - \min(b, d')))(\gamma m - (b - \min(b, d') - m))} \tag{22}$$

Here, Rm is the ratio of cases to controls in the current rule, b is the number of cases represented in that rule, whilst d' is the number of the b case that do not have the potential new genotype. The term, m refers to the minimum value between the number of controls that adhere to the current rule and those in the new genotype and γ is the total number of samples divided by the number of controls for the current rule being built. Finally, all rules generated are tested using a χ^2 test to calculate a statistic and p-value. This two stage approach aims to find any possible interactions quickly before applying a more rigorous statistical test to rank those found.

MPI3SNP

MPI3SNP [38] is specifically designed for fast detection of third order interactions. In order to be most computationally efficient, individuals are represented in a bitwise fashion by their genotype, with a table for cases and one for controls. Using this division probabilities for a combination of SNPs can be quickly ascertained for use in a Mutual Information (MI) equation:

$$I(X, Y) = H(X) + H(Y) - H(X; Y) \tag{23}$$

The MI is calculated by the addition of the entropy of X, the genotype, and Y, the phenotype, followed by the subtraction of the joint entropy. This is repeated exhaustively for all combinations, with the option of using CPU or GPU parallelization in order to further minimize the run time.

2 Supplementary tables

Table S3. Versions of software used

Table S4. Available epistasis detection methods up to 2020

Table S5. EpiGEN Penetrance models with capital genotypes as the major allele. Third dimension indicated as shown in final section for C allele.

Table S6. Key features for each tool
