# Supplementary material for: Evaluating the detection ability of a range of epistasis detection methods on simulated data for pure and impure epistatic models
Source: PLoS One. 2022 Feb 18;17(2):e0263390. doi: 10.1371/journal.pone.0263390 (PMC8856572; doi:10.1371/journal.pone.0263390)
Supplement: S1 File — (ZIP) [file pone.0263390.s001.zip › SuppTab1.pdf]

Two locus 3 x 3 table for allelic combinations, here n is equal to the number of individuals with each genotype

**Minor Allele Dose Per Locus**	**0**	**1**	**2**
**0**	n_{00}	n_{01}	n_{02}
**1**	n_{10}	n_{11}	n_{12}
**2**	n_{20}	n_{21}	n_{22}
