# Supplementary material for: Evaluating the detection ability of a range of epistasis detection methods on simulated data for pure and impure epistatic models
Source: PLoS One. 2022 Feb 18;17(2):e0263390. doi: 10.1371/journal.pone.0263390 (PMC8856572; doi:10.1371/journal.pone.0263390)
Supplement: S1 File — (ZIP) [file pone.0263390.s001.zip › SuppTab2.pdf]

Reduced 2 x 2 table for two loci, here n is equal to the number of individuals with each genotype

Allele Per Locus	Major	Minor
**Major**	$A = 4n_{00} + 2n_{01} + 2n_{10} + n_{11}$	$B = 4n_{20} + 2n_{01} + 2n_{12} + n_{11}$
**Minor**	$C = 4n_{20} + 2n_{21} + 2n_{10} + n_{11}$	$D = 4n_{22} + 2n_{21} + 2n_{12} + n_{11}$
