# Supplementary material for: Evaluating the detection ability of a range of epistasis detection methods on simulated data for pure and impure epistatic models
Source: PLoS One. 2022 Feb 18;17(2):e0263390. doi: 10.1371/journal.pone.0263390 (PMC8856572; doi:10.1371/journal.pone.0263390)
Supplement: S1 File — (ZIP) [file pone.0263390.s001.zip › SuppTab3.pdf]

Versions of software used

Tool	Version	Available
**R**	3.6.0	https://www.r-project.org/
**Java**	11.0.2	https://www.oracle.com/uk/java/technologies/javase/jdk11-archive-downloads.html
**GAMETES**	2.2	https://github.com/UrbsLab/GAMETES
**EpiGEN**	8/10/2020	https://github.com/baumbachlab/epigen
**AntEpiSeeker**	1.0	http://nce.ads.uga.edu/~romdhane/AntEpiSeeker/index.html
**Cassi**	2.5.1	https://www.staff.ncl.ac.uk/richard.howey/cassi/index.html
**CINOEDV**	2.0	https://cran.r-project.org/src/contrib/Archive/CINOEDV/
**epiACO**	1	https://sourceforge.net/projects/epiaco1/files/epiACO.rar/download
**GSS**	02/07/2014	https://github.com/bwgoudey/gwis-stats
**MDR**	3.0.2	https://sourceforge.net/projects/mdr/
**MPI3SNP**	1.0	https://github.com/chponte/mpi3snp/
**PLINK**	1.9b6.17	https://www.cog-genomics.org/plink/
**SNPRuler**	1	https://mybiosoftware.com/snpruler-predictive-rule-inference-epistatic-interaction-detection-genome-wide-association-studies.html
**wtest**	3.2	https://cran.r-project.org/web/packages/wtest/index.html
