# Supplementary material for: Evaluating the detection ability of a range of epistasis detection methods on simulated data for pure and impure epistatic models
Source: PLoS One. 2022 Feb 18;17(2):e0263390. doi: 10.1371/journal.pone.0263390 (PMC8856572; doi:10.1371/journal.pone.0263390)
Supplement: S1 File — (ZIP) [file pone.0263390.s001.zip › SuppTab4.pdf]

Available epistasis detection methods up to 2020

Tool	DOI	Available
**ABCDE**	https://doi.org/10.1515/sagmb-2012-0074	-
**AFT UM-MDR**	https://doi.org/10.5808/GI.2016.14.4.166	-
**AntEpiSeeker**	https://doi.org/10.1038/npre.2012.6994.1	http://nce.ads.uga.edu/~romdhane/AntEpiSeeker/index.html
**AntMiner**	https://doi.org/10.1007/s13258-012-0003-2	https://sourceforge.net/projects/antminer/files/
**BEAM**	https://doi.org/10.1038/ng2110	https://sites.fas.harvard.edu/~junliu/BEAM/
**Bhit**	https://doi.org/10.1186/s12864-015-2217-6	http://digbio.missouri.edu/BHIT/
**BiForce**	https://doi.org/10.1093/nar/gks550	-
**BridGE**	https://doi.org/10.1038/s41467-019-12131-7	http://csbio.cs.umn.edu/bridge
**CAPE**	https://doi.org/10.1371/journal.pcbi.1003270	https://github.com/marta-vidalgarcia/CAPE
**CASSI**	https://doi.org/10.1371/journal.pgen.1002625	https://www.staff.ncl.ac.uk/richard.howey/cassi/installation.html
**CINOEDV**	https://doi.org/10.1186/s12859-016-1076-8	https://cran.r-project.org/src/contrib/Archive/CINOEDV/
**COE**	https://doi.org/10.1089/cmb.2009.0155	http://www.csbio.unc.edu/epistasis/client-coe2.php
**Cox MDR**	https://doi.org/10.1093/bioinformatics/bts415	-
**CSE**	https://doi.org/10.1038/hdy.2014.4	-
**DECMDR**	https://doi.org/10.1093/bioinformatics/btx163	https://drive.google.com/file/d/0B93CxNBXL-MyR1NBZEhMNGRYcUE/view
**Deep Mixed Model**	https://doi.org/10.1186/s12859-019-3300-9	-
**DPEH**	https://doi.org/10.1038/s41598-018-24588-5	-

Continued on next page

Tool	DOI	Available
**DualWMDR**	https://doi.org/10.1002/humu.23951	http://mlda.swu.edu.cn/codes.php?name=DualWMDR
**eCEO**	https://doi.org/10.1093/bioinformatics/btr091	-
**Eigenepistasis**	https://doi.org/10.1186/s12859-017-1488-0	https://github.com/vstanislas/GGEE
**Encore**	https://doi.org/10.1002/gepi.21739	http://insilico.utulsa.edu/index.php/encore/
**epiACO**	https://doi.org/10.1186/s13040-017-0143-7	https://sourceforge.net/projects/epiaco1/files/epiACO.rar/download
**Epiblaster**	https://doi.org/10.1038/ejhg.2010.196	https://www.mybiosoftware.com/epiblaster-1-0-two-locus-epistasis-detection-strategy-gpu.html
**EpiForest**	https://doi.org/10.1186/1471-2105-10-S1-S65	-
**EpiGTBN**	https://doi.org/10.1186/s12859-019-3022-z	http://122.205.95.139/Epi-GTBN/
**epiGWAS**	https://doi.org/10.1371/journal.pone.0242927	https://cran.r-project.org/web/packages/epiGWAS/index.html
**epiMODE**	https://doi.org/10.1371/journal.pgen.1000464	-
**epiNEM**	https://doi.org/10.1371/journal.pcbi.1005496	https://github.com/cbg-ethz/epiNEM
**epistasis**	arXiv:1710.00894v2	https://cran.r-project.org/web/packages/epistasis/epistasis.pdf
**FAACOSE**	https://doi.org/10.1155/2017/5024867	-
**FAM MDR**	https://doi.org/10.1371/journal.pone.0010304	-
**fastChi**	https://doi.org/10.1142/9789812836939_0050	-
**FastLMM**	https://doi.org/10.1038/nmeth.1681	https://fastlmm.github.io/FaST-LMM/
**FCME**	https://doi.org/10.1109/TFUZZ.2019.2914629	https://gitlab.com/yudalinemail/fcmemdr
**FDHE-IW**	https://doi.org/10.3390/genes9090435	-
**FSMDR**	https://doi.org/10.1016/j.artmed.2019.101768	-

Continued on next page

Tool	DOI	Available
**GAIN**	https://doi.org/10.1371/journal.pgen.1000432	https://github.com/insilico
**GeneGeneInteR**	https://doi.org/10.18637/jss.v095.i12	https://bioconductor.org/packages/release/bioc/html/GeneGeneInteR.html
**GenEpi**	https://doi.org/10.1186/s12859-020-3368-2	https://github.com/Chester75321/GenEpi
**GENIE**	https://doi.org/10.1186/1756-0500-4-158	-
**GENN**	https://doi.org/10.1002/gepi.20307	-
**Glide**	https://doi.org/10.1159/000341885	https://github.com/BorgwardtLab/Epistasis-GLIDE
**GMDR**	https://doi.org/10.2174/1389202917666160513102612	http://ibi.zju.edu.cn/software/GMDR/download.html
**GWIS**	https://doi.org/10.1186/1471-2164-14-S3-S10	-
**HS-MMGKG**	https://doi.org/10.2174/1574893614666190409110843	-
**IACO**	https://doi.org/10.1007/978-3-319-42297-8_3	-
**iLOCi**	https://doi.org/10.1186/1471-2164-13-S7-S2	https://www.mybiosoftware.com/iloci-snp-interaction-prioritization-technique-for-detecting-epistasis-in-gwas.html
**IndOR**	https://doi.org/10.1002/sim.5364	http://emily.perso.math.cnrs.fr/IndOR/IndOR/IndOR.html
**Interaction Trees**	https://doi.org/10.1109/ICMLA.2012.114	-
**IOBLPSO**	https://doi.org/10.1155/2015/524821	-
**IPSO**	https://doi.org/10.1371/journal.pone.0037018	-
**JS-MA**	https://doi.org/10.3389/fgene.2020.507038	-
**KCCU**	https://doi.org/10.1186/1471-2156-13-83	-
**KNN-MDR**	https://doi.org/10.1038/nature05911	-
**lampLINK**	https://doi.org/10.1093/bioinformatics/btw418	http://a-terada.github.io/lamplink/
**LINDEN**	https://doi.org/10.1093/nar/gkx505	http://compbio.case.edu/omics/software/linden/
**log-linear MDR**	https://doi.org/10.1093/bioinformatics/btm396	-
**MACOED**	https://doi.org/10.1093/bioinformatics/btu702	http://www.csbio.sjtu.edu.cn/bioinf/MACOED/
**MapReduce**	https://doi.org/10.1080/00207160.2014.1000882	-
**MBMDR PC**	https://doi.org/10.1089/sysm.2019.0003	http://bio3.giga.ulg.ac.be/index.php/software/mb-mdr/
**MBS**	https://pubmed.ncbi.nlm.nih.gov/21346997/	-
**MECPM**	https://doi.org/10.1093/bioinformatics/btp435	https://www.cbil.ece.vt.edu/ResearchOngoingSNP.htm
**MegaSNPhunter**	https://doi.org/10.1186/[illegible]	https://gaow.github.io/g[illegible]
