# Supplementary material for: Evaluating the detection ability of a range of epistasis detection methods on simulated data for pure and impure epistatic models
Source: PLoS One. 2022 Feb 18;17(2):e0263390. doi: 10.1371/journal.pone.0263390 (PMC8856572; doi:10.1371/journal.pone.0263390)
Supplement: S1 File — (ZIP) [file pone.0263390.s001.zip › SuppTab5.pdf]

EpiGEN Penetrance models with capital genotypes as the major allele. Third dimension indicated as shown in final section for C allele.

Joint Dom.	AA	Aa	aa	Joint Rec.	AA	Aa	aa
**BB**	0/0/0	0/0/0	0/0/0	**BB**	0/0/0	0/0/0	0/0/0
**Bb**	0/0/0	0/1/1	0/1/1	**Bb**	0/0/0	0/0/0	0/0/0
**bb**	0/0/0	0/1/1	0/1/1	**bb**	0/0/0	0/0/0	0/0/1
**Modular**	**AA**	**Aa**	**aa**	**XOR**	**AA**	**Aa**	**aa**
**BB**	0/0/0	0/0/0	0/0/0	**BB**	0/0/0	0/0/0	0/0/1
**Bb**	0/0/0	0/0/0	0/0/1	**Bb**	0/0/0	0/0/0	0/0/1
**bb**	0/0/1	0/0/1	0/0/1	**bb**	0/0/1	0/0/1	1/1/0
**Diagonal**	**AA**	**Aa**	**aa**	**C allele**	**AA**	**Aa**	**aa**
**BB**	1/0/0	0/0/0	0/0/0	**BB**	CC/Cc/cc	...	...
**Bb**	0/0/0	0/1/0	0/0/0	**Bb**	CC/Cc/cc	...	...
**bb**	0/0/0	0/0/0	0/0/1	**bb**	CC/Cc/cc	...	...
